# No Relationship between Serum and Salivary β_2_- Microglobulin Levels in A Sample of Adult Diabetic Men with Chronic Kidney Disease without Renal Replacement Therapy

**Published:** 2014-05-25

**Authors:** Ahmadreza Assareh Assareh, Habib Haybar, Hosein Malekzadeh, Leila Yazdanpanah, Mohammadreza Bozorgmanesh

**Affiliations:** 1 Cardiovascular Research Center, Golestan Hospital , Ahvaz Jundishapur University of Medical Science, Ahvaz, Iran; 2 Department of Oral Medicine, Ahvaz Jundishapur University of Medical Science, Ahvaz, Iran; 3 Diabetes Research Center, Ahvaz Jundishapur University of Medical Science, Ahvaz, Iran; 4 Prevention of Metabolic Disorders Research Center, Research Institute for Endocrine Sciences (RIES), Shahid Beheshti University of Medical Sciences, Tehran, Iran

**Keywords:** β_2_-Microglobulin, Chronic Kidney Disease, Saliva

## Abstract

**Objective:**

Β_2_-microglobulin (β_2_M) associated amyloidosis is an inevitable complication of chronic kidney disease (CKD). Testing β_2_M in the blood is invasive and expensive. On the other hand, oral fluid is a perfect medium to be explored for public health and disease surveillance. However, it has never been studied if salivary concentration of β_2_M reflects its concentration in the serum. The current study; therefore, aimed to examine the relationship between salivary and serum β_2_M in a sample of adult diabetic men with CKD.

**Materials and Methods:**

Among diabetic patients referred to the Nephrology Department of The Golestan Hospital of Ahvaz due to CKD, 40 men not requiring renal replacement therapy were consecutively recruited for this cross-sectional study. Patients were excluded if they had any disease or were using any drugs that might affect the oral mucosa or saliva. The concentration of β_2_M was measured in both serum and saliva. The correlation between serum and salivary β_2_M was measured by calculating spearman’s ρ.

**Results:**

The Spearman’s ρ for correlation between serum and salivary β_2_M was -0.017 (p=0.917), indicating lack of correlation. Serum and salivary creatinine (Spearman’s ρ=0.54; p value<0.001) as well as serum and salivary urea nitrogen levels (Spearman’s ρ=0.39; p value=0.014) were correlated.

**Conclusion:**

Salivary β_2_M levels poorly agreed with serum β_2_M levels, and thus may not be used as a surrogate for serum β_2_M in CKD patients who did not require replacement therapy.

## Introduction

The number of patients with chronic kidney disease (CKD) is rising rapidly worldwide (1), and CKD is increasingly recognized as a global public health burden (2, 3). Diabetes mellitus is the most common cause of end stage renal disease (ESRD) in many countries, and it has been estimated that 366 million people will have diabetes mellitus by 2030 (4). Furthermore, diabetes and CKD exhibit synergistic associations with cardiovascular disease and premature mortality (5). There is now a plethora of evidence to indicate that CKD can be detected using simple laboratory tests, and that timely treatment can prevent or delay complications of decreased kidney function, slow the progression of kidney disease, and reduce the risk of cardiovascular disease (CVD). These advances must be translated to simple and applicable public health measures. Developing a public health policy to improve outcomes entails the understanding the relationship between CKD and other chronic diseases (2).

Kidney failure requiring renal replacement therapy (dialysis or renal transplantation) is the most visible outcome of CKD. However, CVD frequently complicates CKD and individuals with CKD are more likely to die of CVD than to develop kidney failure (6-10), this disease could potentially be treated and prevented among patients with CKD (11, 12).

Β_2_-microglobulin (β_2_M) associated amyloidosis is considered an inevitable complication of chronic hemodialysis (13). β_2_M constitutes a light chain of the class I major histocompatibility complex. Widely distributed in nucleated cells in the body, β_2_M is especially rich in immunocompetent cells, such as lymphocytes or monocytes. Various stimuli cause substantial amounts of the molecule to be shed into the circulation (14). Circulating β_2_M is filtered through the glomeruli and is reabsorbed and metabolized in the proximal tubules of the kidneys (14). Therefore, the plasma concentration of β_2_M is largely affected by the glomerular filtration rate (GFR) of the kidneys. Β_2_M has recently been shown to be related to risk factors of the atherosclerosis, coronary heart disease (15, 21), cardiovascular (20) and total mortality (15, 21).

Due to peripheral venous access difficulty in CKD patients, the plasma concentration of β_2_M microglobulin is not applicable to them. Saliva, as a unique fluid of diagnostic medium, has advanced exponentially in the last decade. While testing β_2_M in the blood is invasive, there are less invasive methods available to test β_2_M in saliva. The ability to measure and to monitor a wide range of molecular components in saliva and to compare their levels to plasma components levels have made possible to study microbes, chemicals, and immunologic markers (22, 23).

It has, however, not been studied if salivary concentration of β_2_M reflects its concentration in the serum. If it has been the case, salivary β_2_M would have provided a unique opportunity as a simple chair-side tool for periodical assessment of patients with CKD. The current study, therefore, aimed to examine the relationship between salivary and serum β_2_M in a sample of adult diabetic men with CKD.

## Materials and Methods

### Patients and design

Among diabetic patients referred for CKD to the Nephrology Department of the Golestan Hospital of Ahvaz, Ahvaz, Iran, 40 male were consecutively recruited for the current cross-sectional study. Patients were excluded if they had parageusia (4), were smoker (2) or using any drugs having affected their salivary flow or content, or if there was any other evidence of a systemic disease affecting the oral mucosa or saliva. Patients who required renal replacement therapy were also excluded. Renal replacement therapy is initiated once patients have stage 5 disease or signs of uremia, including lack of appetite, nausea, vomiting, acidosis, hyperkalemia, or fluid overload (24).

### Measurements

After a period of 8 to 12-hour overnight fasting, non-stimulated saliva samples were taken from all participants. Participants were instructed not to speak during the saliva collection period. To prevent changes in salivary composition during a 24-hour period, they were instructed not to eat, drink, and use toothbrush, toothpaste, or mouthwash since 2 hours before sample collection. Participants were asked to spit their saliva into a test tube 5 minutes after they washed and rinsed their mouth with water. The sampling was then continued until 10 ml of saliva specimen was collected. A Blood sample (5 ml) was also taken from all participants via venipuncture. Serum and salivary β_2_M was measured using Minineph Human Kit (Binding Site Co., UK), while urea and creatinine in saliva and blood samples were measured using Pars Azmun Kit (Pars Azmoon Inc., Tehran, Iran).

### Definitions of terms

GFR was estimated using Cockcroft-Gault equation (25):

CCr=(140-age)*Weight*GFPCr*72

The gender correction factor (GF) is 1.00 for men and 0.85 for women. We did not used the equation derived from the Modification of Diet in Renal Disease (MDRD) study because it was not validated for diabetic kidney disease (2).

CKD was defined as kidney damage for ≥3 months and/or GFR <60 ml.minute^-1^ per 1.73 m^2^ for ≥3 months with or without kidney damage. Kidney damage was defined as structural or functional abnormalities of the kidney, initially without decreased GFR (26). ESRD was ascertained in participants with GFR <15 ml.minute^-1^ (27).

Xerostomia was defined clinically. According to the medical literature, dryness of the cheek mucosa was determined by visual examination, palpation and adherence degree of mucosal surface using a wooden spatula (28). Hypertension, diabetes, smoking, and parageusia were diagnosed by self-reporting.

### Statistical analysis

Data are presented as either mean (SD) or frequency (%) for continuously-and categoricallydistributed variables, respectively. Median [interquartile range (IQR)] used for continuously distributed variable revealed that distribution was not normal.

The linear regression model was used to examine the statistical significance of the association between serum β_2_M, while controlling for age. We used Spearman’s ρ as a coefficient of concordance between serum β_2_M and salivary β_2_M, of concordance between serum urea nitrogen and salivary urea nitrogen, and of concordance between serum creatinine and salivary creatinine.

Instead of using arbitrary predetermined cutpoints to capture nonlinear aspects of association, we used restricted cubic splines functions of the salivary β_2_M to represent their continuous relationship with the serum β_2_M, so that the relationships were meaningfully in accordance with substantive background knowledge. Splines functions, as phrased by Harrell, are "piecewise polynomials within the intervals of a variable that are connected across different intervals of that variable" (29). Restricted cubic splines function enabled us to use flexibly model continuous predictors (salivary β_2_M), while allowing us to control over the excessive instability and tendency of spline functions in order to generate artifactual and uninterpretable features of a curve. Multivariate restricted cubic splines were used with 4 knots defined at 5^th^, 25^th^, 75^th^, and 95^th^ percentiles (29). In variable selection, we dropped a variable if its removal caused a non-significant increase in deviance.

We used several criteria to compare the overall predictive values of alternative models. In addition, the outcome variables are referred to goodness-offit when a model effectively describes them. We used following measures of goodness-of-fit.

1. *Deviance* compared the fit of the saturated model to the fitted model. This was a small value if the model was good. For purposes of assessing the significance of non-linear terms, the values of D with and without the non-linear terms were compared by likelihood ratio test.
2. *Akaike information criterion* (AIC) was used to account for complexity. Difference in AIC >10 was considered significant (30).

### Ancillary analysis

In order to apply ancillary analysis, we examined the predictive ability of the serum and salivary β_2_M for renal failure. Owing to high prevalence rate of renal failure in the study sample, the odds ratios obtained from the logistic regression model would be excessively large. When a study outcome is rare, the odds ratio estimate of causal effects will approximately be the risk ratio. However, if a study outcome is common (>10%), the odds ratio will be further from 1 than the risk ratio (31, 32). We, thus, used the Cox proportional hazard regression model with time as constant variable to approximate risk ratio in order to avoid the renal failure for increasing levels of β_2_M.

The significance levels for selection of spline functions by backward elimination were set at 0.1. For salivary β_2_M, however, we set the significance level at unity, forcing it into the model, leaving the others to be selected or not. For the rest of the analyses, we set the statistical significance level at a two-tailed type I error of 0.05. All statistical analyses were performed using STATA version 12 (STATA, College Station, Texas, USA). All applicable institutional and governmental regulations concerning the ethical use of human volunteers were followed during this research. Informed written consent was obtained from all participants, while the Ethical Committee of the Ahvaz Jundishapur University of Medical Sciences approved this study.

## Results

Mean age of the participant was 60.6 (14.0) years. ESRD was documented in 15 out of 40 participants (38.5%). Baseline characteristics of participants have been presented in table 1. The median and IQR of serum and of salivary β_2_M were 9.20 (0.44) and 6.23 (3.78), respectively. All patients were at taking either angiotensin converting enzyme inhibitor or angiotensin II receptor antagonist.

As shown in the table 2, none of the studied variables were associated with serum β_2_M, while showing a p>0.1.

**Table 1:** Baseline characteristics of participants


Variable	Median (IQR)

**Age (Y) **	61.00 (13.50)
**Height (cm)**	170.00 (4.00)
**Weight (kg)**	80.00 (19.00)
**Body mass index (kg.m^-2^)**	27.07 (6.32)
**Blood urea nitrogen (mg.dl^-1^)**	48.00 (38.00)
**Serum creatinine (mg.dl^-1^)**	3.30 (5.55)
**Serum β_2_-microglobulin (mg.dl^-1^)**	9.20 (0.44)
**Salivary urea nitrogen (mg.dl^-1^)**	13.00 (43.00)
**Salivary creatinine (mg.dl^-1^)**	0.25 (0.40)
**Salivary β_2_-microglobulin (mg.dl^-1^)**	6.23 (3.78)


**Table 2:** Association of β_2_M with different variables


Variable	Regression coefficient (β)^1^	SE	Z	p value	95% CIs	

**Body mass index (kg.m^-2^)**	0.70	1.56	0.44	0.663	-2.47	3.86
**Serum urea nitrogen (mg/dl)**	7.62	10.30	0.74	0.461	-13.27	28.50
**Serum creatinine (mg/dl)**	1.45	1.09	1.34	0.194	-0.75	3.66
**Salivary urea nitrogen (mg/dl)**	4.43	9.71	0.46	0.651	-15.25	24.11
**Salivary creatinine (mg/dl)**	0.09	0.12	0.77	0.453	-0.15	0.34
**Salivary β_2_-microglobulin (mg/dl)**	0.71	0.71	1.01	0.322	-0.72	2.15


Effect size denotes age-adjusted effect of a one-unit increase in β_2_M on varying variables. It was obtained from age-adjusted linear regression models (coefficient of regression).

**Fig 1:**
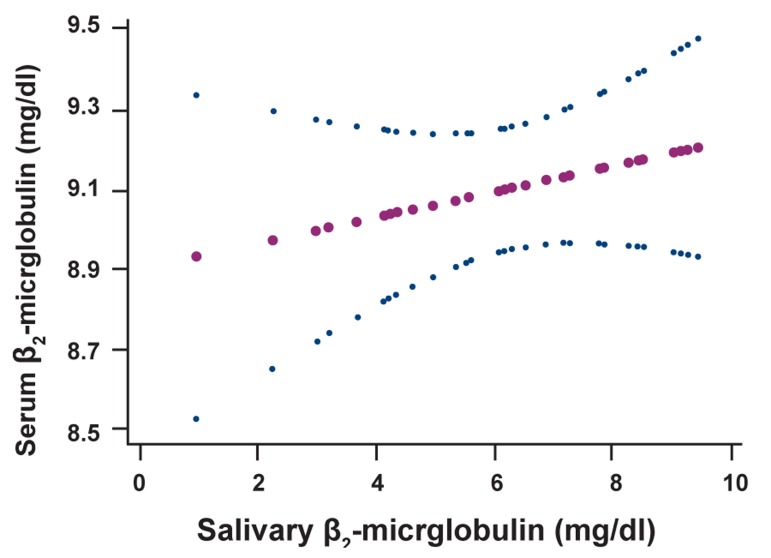
The nonlinear association between serum and salivary β_2_M.

The Spearman’s ρ for correlation between serum and salivary β_2_M was -0.017 (p=0.917), indicating lack of correlation. We also investigated the nonlinear association between serum and salivary β_2_M and found no evidence of nonlinearity as is depicted in the figure 1.

Serum and salivary creatinine (Spearman’s ρ=0.54; p<0.001) as well as serum and salivary urea nitrogen levels (Spearman’s ρ=0.39; p=0.014)) were correlated.

Increasing concentrations of serum β_2_M (RR=1.44, 95% CIs: 0.41-5.05; p=0.571) and salivary β_2_M (RR=1.06, 95% CIs: 0.82-1.36; p=0.671) were associated with non-significant increased risk of ESRD. We also investigated the nonlinear association between serum and salivary β_2_M. The incorporating natural cubic spline functions of the Salivary β_2_ Macroglobulin (nonlinear linear term only (likelihood ratio test χ^2^=5.4; p=0.134). As shown in the table 3, it is evident that there are no significant improvements in the values of AIC.

**Table 3:** Nonlinear versus linear association between salivary β_2_ macroglobulin and end stage renal disease


		β coefficient	SE	Z	p value	95% CIs	

**Nonlinear model**							
**Salivary β_2_ macroglobulin **	Standardized function	0.09	0.08	1.16	0.26	-0.07	0.25
	Orthogonalized basis 1	0.10	0.08	1.30	0.20	-0.06	0.26
	Orthogonalized basis 2	-0.05	0.08	-0.65	0.52	-0.21	0.11
	Orthogonalized basis 3	0.14	0.08	1.76	0.09	-0.02	0.30
	Age (Y)	0.01	0.01	1.24	0.22	0.00	0.02
	Intercept	8.65	0.37	23.26	0.00	7.90	9.41
**Akaike information criteria**	61.19						
**Deviance **	49.2						
**Linear model**							
**Salivary β_2_ macroglobulin **	Linear function	0.04	0.04	1.01	0.32	-0.04	0.11
	Age (Y)	0.00	0.01	0.67	0.51	-0.01	0.02
	Intercept	8.63	0.47	18.54	0.00	7.69	9.58
**Akaike information criteria**	60.77						
**Deviance **	54.8						
**Nonlinear vs. linear likelihood ratio test χ^2^ (p value)**	5.6 (0.134)						


## Discussion

Using a blood sample of adult diabetic men with CKD, we examined the concordance between serum and salivary β_2_M and observed that salivary β_2_M levels poorly agreed with serum β_2_M levels, and thus may not be used as a surrogate for serum β_2_M. We observed, however, a moderate correlation between serum and salivary levels of creatini ne and urea.

Our finding of interest was that both salivary and serum levels of β_2_M predicted the presence of ESRD, although the contributions failed to achieve statistical significance. The degree of accumulation of β_2_M in patients undergoing hemodialysis have been previously observed to depend on the loss of renal excretory function (33). In patients with chronic renal failure, β_2_M levels have been observed to parallel an increase in serum creatinine. A dramatic decrease in beta β_2_M levels have been reported to be correlated with improvement in GFR (34).

Zhang et al. (35) have argued that for clinical applications, such as monitoring health status, disease onset and progression, and treatment outcome, there are following three necessary prerequisites:

1. A simple method for collecting biologic samples, ideally noninvasively.
2. Specific biomarkers associated with health or disease.
3. A technology platform to rapidly utilize the biomarkers.

β_2_M has been demonstrated to be a major prognosticator of mortality in hemodialytic patients, independent of hemodialysis length, diabetes, malnutrition and chronic inflammation, suggesting the clinical importance of lowering and periodical monitoring of serum β_2_M in these patients (36). However, venipuncture of the patients with CKD is an exhausting task to accomplish both for patients and health care providers. It is a highly desired skill in health care promotion and delivery to monitor health status, disease onset and progression, and treatment outcome through nonaggressive methods. Saliva is considered a complete medium to be explored for health and disease inspection (37). Saliva is appealing in that thereof taking samples does not require invasive procedure. It is commonly considered as the 'mirror of the body', and can be a perfect alternate method for clinical diagnostic (35). Utilizing easily accessible saliva for evaluating CKD may enable frontline care providers to become more involved and proactive in the management of CKD, facilitating a new way in order to focus on early detection and targeted interventions of vulnerable persons.

Achieving the goal of salivary diagnostics needs two prerequisites to be completed first:

1. Identification of specific biomarkers associated with a health or disease state.
2. The development of technologies that can discriminate between the biomarkers.

Recently, National Institute of Dental and Craniofacial Research has set a goal of using saliva as the diagnostic medium to evaluate the health or disease status of patients. This attempt could be looked upon as an ideal opportunity to optimize state-of-the-art saliva-based biosensors for salivary biomarkers that discriminate between diseases (37).

We observed a moderate correlation between serum and salivary levels of creatinine and urea. The concentration of salivary creatinine has been documented to be 10-15% of serum creatinine concentrations in healthy people. It has been, however, argued that this proportion may not hold among patients with renal disease. It has been shown that salivary creatinine estimations may be used to identify subjects with serum creatinine concentrations above 120 mmol.l-1 (38). Goll and Mookerje have pointed that "in hemodialysis patients, concentration of serum creatinine and uric acid is correlated with those in simultaneously drawn unstimulated whole saliva before and after dialysis" (39). The same findings have also been observed among patients with moderate renal failure not requiring the chronic hemodialysis. Use of whole saliva in this setting may preclude the iatrogenic component in anemia by cutting down the frequency of venipuncture; this could be of greater importance to young patients. On the other hand, it has been argued that salivary composition in patients with CKD varies by the stage of renal failure (40).

### Limitations

We did not use immunoassay for measurement of β_2_M. Furthermore, levels of serum β_2_M did not vary among participants in the current study. We failed to demonstrate any association between increased levels of serum or salivary β_2_M and ESRD. Wide confidence intervals indicated that our sample size probability did not have enough statistical power to capture the trivial associations observed.

## Conclusion

Using a blood sample of adult diabetic men with CKD, we examined the concordance between serum and salivary β_2_M and observe that salivary β_2_M levels poorly agreed with serum β_2_M levels, and thus may not be used as a surrogate for serum β_2_M in this highly selected subgroup of patients. Future prospective studies with larger sample size will be required to investigate whether β_2_M can predict development of ESRD or not. We observed, however, a moderate correlation between serum and salivary levels of creatinine and urea. The clinical relevance of these findings remains to be illustrated.
